# Characterizing Cardiotoxicity of FDA-Approved Soft Tissue Sarcoma Targeted Therapies and Immune Checkpoint Inhibitors: A Systematic Review

**DOI:** 10.3390/cancers17050827

**Published:** 2025-02-27

**Authors:** Mustafa Houmsse, Andrew Muskara, Damaris Pasca, Arnab Roy, Sana Sughra, Sanam Ghazi, Daniel Addison, Marium Husain

**Affiliations:** 1 College of Medicine, Northeast Ohio Medical University, Rootstown, OH 44272, USA; mhoumsse@neomed.edu (M.H.); amuskara@neomed.edu (A.M.); 2 College of Graduate Studies, Northeast Ohio Medical University, Rootstown, OH 44272, USA; dpasca@neomed.edu (D.P.); aroy3@neomed.edu (A.R.); 3 Cardio-Oncology Program, Division of Cardiology, The Ohio State University Wexner Medical Center, Columbus, OH 43210, USA; sana.sughra@osumc.edu (S.S.); sanam.ghazi@osumc.edu (S.G.); 4 Division of Cancer Control and Prevention, James Cancer Hospital, The Ohio State University, Columbus, OH 43210, USA; 5 Division of Medical Oncology, Department of Internal Medicine, The Ohio State University Wexner Medical Center, Columbus, OH 43210, USA

**Keywords:** cardio-oncology, soft tissue sarcoma, targeted therapies, immunotherapies, major adverse cardiovascular events

## Abstract

**Background**: Soft tissue sarcomas (STS) are aggressive cancers that show increasing response to novel targeted-therapies and immune-checkpoint-inhibitors. Despite anecdotal reports of cardiovascular adverse events (AEs) and major adverse cardiovascular events (MACE) potentially hindering their utility, the true cardiotoxic profile of these novel-therapies in STS has been largely understudied. **Methods**: We assessed the incidence and severity of AEs and MACE of contemporary FDA-approved targeted and immune-based therapies for STS, using data from landmark clinical trials supporting FDA-approval. We also analyzed data from the FDA adverse-event-reporting-system-(FAERS) for FDA-approved STS targeted and immune-based therapies for comparative real-world validation. **Results**: Overall, 12 clinical trials supporting FDA-approval of STS targeted-therapies and immune-checkpoint-inhibitors, incorporating 1249 patients, were identified. These clinical trials revealed 751 AEs including, hypertension (382, 50.87%), atrial fibrillation (3, 0.40%), myocardial infarction (2, 0.27%), cardiac failure (congestive included) (9, 1.20%), and cardiac failure (heart failure included) (7, 0.93%). Compared to placebo, those treated saw higher MACE (OR: 3.27, *p* < 0.001). The FAERS data showed 489 reported AEs including hypertension (275, 56.24%), atrial fibrillation (31, 6.34%), myocardial infarction (15, 3.07%), and cardiac failure (congestive included) (30, 6.13%). Programmed death-ligand 1 (PD-L1) inhibitors had the highest probability of AEs (0.65, 1.17), followed by tyrosine kinase inhibitors (0.66, 0.11), tropomyosin receptor kinase inhibitors (0.25, 0.13), mammalian target of rapamycin inhibitors (0.21, 0.09), and enhancer of zeste homologue 2 inhibitors (0.11, 0.06). Proportions were calculated from the samples in clinical trials supporting FDA-approval and FAERS, respectively. **Conclusions**: In this investigation, contemporary FDA-approved therapies for STS are associated with increased risk of AEs

## Simple Summary

Targeted therapies and immune checkpoint inhibitors are increasingly used for soft tissue sarcoma (STS), yet their cardiovascular risks remain unclear. This systematic review evaluates cardiovascular adverse events (AEs) associated with FDA-approved STS therapies using clinical trial data and FAERS reports. Hypertension, atrial fibrillation, and heart failure were frequently reported, with real-world data suggesting a higher incidence than clinical trials. Notably, PD-L1 inhibitors had the highest probability of AEs. These findings highlight the need for vigilant cardiovascular monitoring in STS patients receiving novel therapies to improve long-term safety and outcomes

## 1. Introduction

Nearly 35,000 patients in the United States are diagnosed with soft tissue sarcoma (STS) every year [1,2]. STS poses significant challenges in treatment due to its aggressive nature, tendency to infiltrate nearby tissues, recurrence, and metastasis [3]. Traditional treatment modalities for soft tissue sarcomas include surgery, radiation, or chemotherapy. Commonly used chemotherapy drugs are Doxorubicin, Ifosfamide, Dacarbazine, Gemcitabine, and Trabectedin [4,5]. However, the effectiveness of these treatments is often limited [6]. Consequently, there has been a push to explore new therapeutic strategies such as targeted and immune-based therapies. In available trials, many therapies have been linked with dramatic anticancer efficacy. For example, Atezolizumab, an immune checkpoint inhibitor was effective at inducing sustained responses in almost one third of patients with advanced alveolar soft part sarcoma (ASPS), which is a rare soft-tissue sarcoma with a poor prognosis [7].

Despite these observations, there is relatively limited data on potential disease-specific effects of targeted and immunotherapies [8]. Emerging anecdotal reports have suggested an increase in potentially unintended events, particularly inclusive of cardiovascular sequelae [9]. For example, in a recent landmark trial of immunotherapy-treated STS patients, nearly 23% developed cardiotoxic arrhythmias [7]. This is concerning, as many STS patients require multiple courses or lines of therapy [10]. Yet, given the isolated nature of these available data, whether cardiotoxicity is frequently seen among STS patients or has an effect on long-term outcomes is unknown.

Furthermore, there is a growing body of evidence suggesting that adverse cardiovascular events associated with anticancer therapies may be more common than initially anticipated. A study, including data from 189 trials, evaluated the total number of reported CVD and MACE events in clinical trials for all FDA-approved cancer therapies from 1998 to 2018. It was found that for the MACE events the reported incidence rate of 542 per 100,000 person-years was significantly less than the incidence rate of 1408 per 1000,000 person-years that was reported among similar-aged non-cancer trial subjects [11]. This highlights an intriguing discrepancy that exists within the reporting process for FDA approval when testing the efficacy and safety of cancer therapies. This discrepancy may be attributed to differences in patient selection, as clinical trials often exclude individuals with pre-existing cardiovascular conditions. Additionally, underreporting of adverse events, variations in event definitions, and shorter follow-up durations in clinical trials may contribute to the lower reported incidence rates [11]. In the current investigation, both FDA-approved targeted therapies and immune checkpoint inhibitors are considered in order to fill a crucial gap of knowledge in the literature and in practice [12]. As anthracyclines are traditionally known to be a potential limiting factor in sarcoma treatment, this study provides needed context to the current landscape and provides clinicians and patients with more updated context to the risk of CVD and other serious events following treatment [13].

## 2. Methods

**Figure 1:**
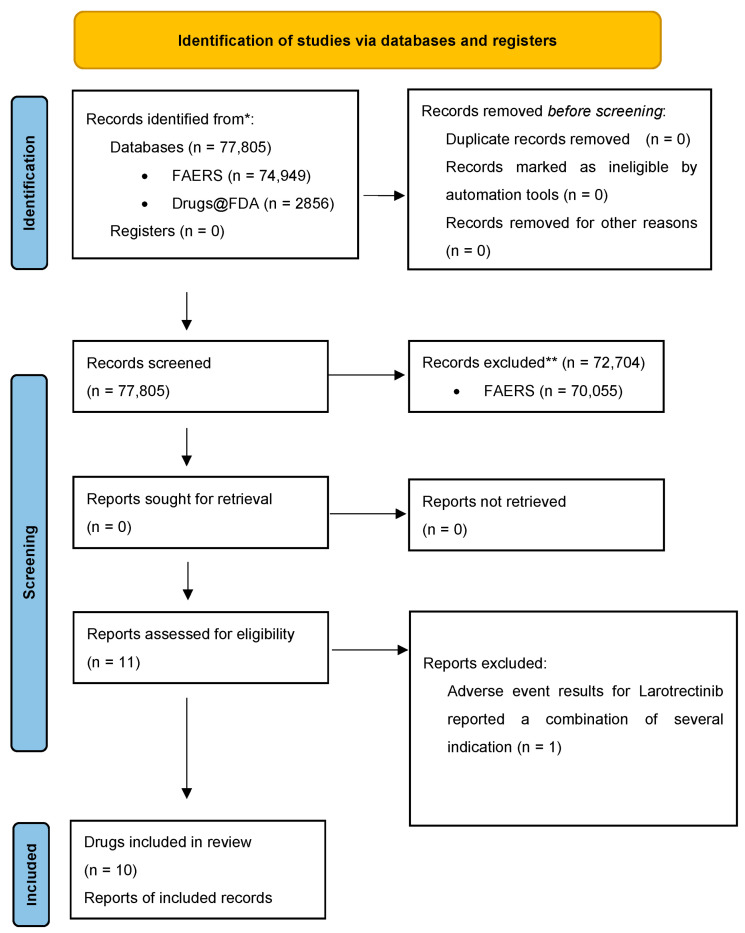
Flowchart of record selection and screening. * Initial reported adverse events in FAERS for cancer therapies regardless of indication, clinical trials were identified to be supporting for FDA approval. ** Number of reported adverse events that were not indicated for soft tissue sarcoma.

This review was performed in accordance with the PRISMA (Preferred Reporting Items for Systematic Reviews and Meta-Analyses) guidelines and has not been registered. Leveraging the Drugs @ FDA database, we performed a manual search of all anticancer therapies given new drug application (NDA) approvals from 2001 to 2024 [14]. Data for cardiovascular adverse events (AEs) of FDA approved soft tissue sarcoma targeted therapies and immune checkpoint inhibitors were obtained from clinical trials supporting FDA-approval and the FDA Adverse Event Reporting System (FAERS). Adverse event (AE) data from clinical trials supporting FDA-approval and AE data from FAERS were collected and analyzed separately. Clinical trials supporting FDA-approved novel therapies were identified from the clinical studies section of each therapy’s FDA drug label. Results from clinical trials and FAERS were recorded in several categories which included case ID, drug indication, and AEs. The AEs were broken down into two sections: serious and non-serious, consistent with clinical trial results reporting. The flow chart of record selection and screening is shown in Figure 1.

The reported data from FAERS was then pooled and tabulated to convey the incidence of cardiac related AEs amongst the chosen targeted therapies and immune check point inhibitors. To account for varying sample sizes between drug classes, the proportion was calculated for each drug class from both FDA supporting studies and FAERS. We also reported the percentage incidence of total cardiac AEs reported for the entire sample size of the respective clinical trial. The AEs having higher associated cardiotoxicity risk or high cardiac AE potential were also included in the figures [15].

### 2.1. Clinical Trial Inclusion and Exclusion Criteria

Landmark sarcoma trials, from 2006 to 2023, supporting contemporary treatment were identified [1]. The studies used in analysis were carefully defined to ensure relevance and consistency in assessing the efficacy and safety of targeted therapies and immune checkpoint inhibitors for soft tissue sarcoma. Clinical trials reported in the clinical studies section of the FDA drug label for each therapy with a soft tissue sarcoma indication were considered for inclusion. These trials were chosen because their results were used to determine the safety and effectiveness of each therapy for FDA-approval.

The clinical trials in the clinical studies section of the FDA drug label without a soft-tissue sarcoma indication were excluded from the analysis. Additionally, clinical trials with combined soft-tissue sarcoma and non-soft-tissue sarcoma indications were excluded. Furthermore, trials lacking a history of cardiovascular disease within their respective exclusion criteria were omitted, as the potential cardiotoxic effects of novel treatments are of particular concern in the context of soft tissue sarcoma therapy. Additionally, studies that evaluated conventional cancer therapies, such as radiation and chemotherapy, were excluded to maintain the scope of the analysis on novel immune checkpoint inhibitors and targeted drugs.

These inclusion and exclusion criteria were adhered to by two independent reviewers. The analysis aimed to provide a comprehensive and rigorous assessment of the efficacy and safety profile of novel therapeutic interventions for soft tissue sarcoma to ensure the relevance and reliability of the findings.

### 2.2. Variables Considered in Data Analysis

When collecting data from FDA supporting studies and FAERS, several categories of cardiovascular, or related adverse events were quantified by each reviewer. Major adverse cardiovascular events (MACE) were considered as any incidences of cardiovascular death, myocardial infarction, stroke, coronary revascularization, or heart failure. Arrhythmias in this analysis included atrial flutter, atrial fibrillation or ventricular fibrillation. Any incidence of embolism, thrombosis, and edema were also included as they may be related to or indicative of cardiovascular health. Additional variables such as significant episodes of increased or decreased heart rate, blood pressure, hypertension, myocardial infarction, cardiac arrest, transient ischemic attacks, and pericardial effusion were also included for analysis

### 2.3. Addressing Risk of Bias

Each reviewer was given specific events to include in data collection such as MACE, arrhythmia, and hypertension. Each term was given a clear description for each reviewer to reference when gathering data in order to prevent misinterpretation of the classification of each respective event; for example, MACE events included any incidences of atrial fibrillation, cardiovascular death, myocardial infarction, stroke, coronary revascularization, or heart failure. All analyses were performed with SAS software version 9.4 (SAS Institute, Cary, NC, USA), and the statistical tests were 2-sided with statistical significance evaluated at the α = 0.05 significance level.

## 3. Results

Overall, there were 12 later-phase clinical trials, enrolling 1249 patients, supporting FDA-approval of targeted and immunotherapies for STS (Supplemental Table S1) [7,16,17,18,19,20,21,22,23,24,25,26]. Among the clinical trials supporting FDA approval of targeted therapies and immune checkpoint inhibitors for soft tissue sarcoma, a total of 751 adverse cardiovascular events were reported. Hypertension (382, 50.87%) was the most common cardiovascular-related adverse event, followed by peripheral edema (119, 15.85%), hypotension (41, 5.46%), left ventricular dysfunction (29, 3.86%), and decreased ejection fraction (25, 3.32%) (Table 1).

Within FAERS, a total of 489 adverse cardiovascular events were reported. Hypertension (275, 56.24%) was the highest reported adverse cardiovascular event across all therapies of interest, followed by atrial fibrillation (31, 6.33%) and cardiac failure (congestive included) (30, 6.13%) (Table 2).

### 3.1. Characteristics of Adverse Cardiovascular Events Reported in the FAERS Database

To adjust for sample size variability, the probabilities to experience adverse events were calculated for each drug class within the FDA clinical trials and FAERS, using incidence rates expressed as the total number of adverse events per corresponding denominator: trial subjects for clinical studies, or cumulative reports for FAERS. Within the FAERS reporting system, 12,337 total adverse events were reported. Of those reported events, 489 were identified as adverse cardiovascular events. Programmed death-ligand 1 inhibitors had the highest probability to experience adverse cardiovascular events of (1.17), followed by tropomyosin kinase inhibitors (0.13), tyrosine kinase inhibitors (0.11), mammalian target of rapamycin inhibitors (0.09), and enhancer of zeste homolog 2 inhibitors (0.06).

### 3.2. Characteristics of Adverse Cardiovascular Events Reported in FDA Clinical Trials

Within FDA supporting clinical trials, tyrosine kinase inhibitors had the highest probability to experience a cardiovascular adverse event (0.66), followed by programmed death-ligand 1 inhibitors (0.65), tropomyosin kinase inhibitors (0.25), mammalian target of rapamycin inhibitors (0.21), and enhancer of zeste homolog 2 inhibitors (0.11). Comparisons of cardiovascular events between treatment and placebo were made among FDA supporting clinical trials with placebo arms, which included tyrosine kinase inhibitors Pazopanib, Regorafenib, Ripretinib, and Sunitinib. Overall, patients treated in the intervention arms of these trials saw an increase in the probability of a reported cardiovascular event (OR: 3.27, *p* < 0.001); Supplemental Tables S2 and S3.

### 3.3. Incidence of Adverse Cardiovascular Events by Therapy

Sunitinib and Ripretinib were considered to have the highest incidences of adverse cardiovascular events in FDA supporting clinical trials and FAERS, respectively. Larotectinib and Tazemetostat had the least number of adverse cardiovascular events reported from the same source. Pulmonary embolism was only found in the analysis of adverse events reported in FAERS, but was not reported in the published literature or clinical trials. Among FDA supporting clinical trials with placebo arms, Pazopanib had the highest probability of a reported cardiovascular event compared to placebo (OR: 5.34, *p* < 0.001).

### 3.4. Hypertension

Hypertension was found to be the most commonly reported adverse event across all drugs in the FAERS reporting system, with an incidence of 56.24% of the total 489 reported CVDS. Ripretinib had the most individual reports (175, 35.79%), followed by Regorafenib (53, 10.94%), and Pazopanib (36, 7.36%) (Table 2). Among FDA supporting studies, hypertension was not only the most reported but took up the majority of the 751 reported adverse cardiovascular events with an incidence of 50.87%. Regorafenib had the most individual reports (125, 16.64%), followed by Sunitinib (115, 23.52%) and Pazopanib (101, 13.45%) (Table 1). Furthermore, among FDA supporting clinical trials with placebo arms, hypertension was the adverse cardiovascular event with the highest probability in the treatment arm compared to the placebo arm for all drugs (Pazopanib OR: 1.39, *p* < 0.001; Regorafenib OR: 1.35, *p* = 0.134; Sunitinib OR: 1.31, *p* < 0.001; Ripretinib OR: 1.03, *p* = 0.139).

### 3.5. Atrial Fibrillation

Atrial fibrillation was found to have an incidence of 6.34% among the 489 reported adverse cardiovascular events from the FAERS reporting system. Ripretinib (17, 3.48%) had the highest number of reports, followed by Regorafenib (9, 1.84%) (Table 2). Among FDA supporting studies, atrial fibrillation showed an incidence of only 0.40% between Sunitinib (2, 0.27%) and Regorafenib (1, 0.13%) (Table 1).

### 3.6. Cardiac Failure

Cardiac failure (congestive included) was also found to have an incidence of 6.13% in the 489 reported adverse cardiovascular events from the FAERS reporting system. As seen in atrial fibrillation and hypertension, Ripretinib also had the most reports for combined cardiac failure (9, 1.84%) along with Pazopanib (9, 1.84%), followed by Regorafenib (7, 1.43%) (Table 2). Among FDA supporting studies, combined cardiac failure showed an incidence of 1.20%. Sunitinib had the highest number of reports (6, 0.80%), followed by Entrectinib (2, 0.27%) and Ripretinib (1, 0.13%) (Table 1).

### 3.7. Myocardial Infarction

Myocardial infarction showed an incidence of 3.07% among the 489 reported adverse cardiovascular events from the FAERS reporting system. Ripretinib (9, 1.84%), Regorafenib (3, 0.61%), and Pazopanib (3, 0.61%) were the sole drugs with myocardial infarction (Table 2). Among FDA supporting studies, Sunitinib (2, 0.27%) was the only drug to report myocardial infarction.

## 4. Discussion

In this evaluation of the incidence and implications of cardiac events in patients requiring contemporary anti-cancer treatments for STS, a total of 1240 adverse cardiovascular events were reported in both data sources. Hypertension, arrhythmias, and heart failure were the most commonly reported cardiac events. In clinical post-marketing analysis, atrial fibrillation appeared relatively more common than reported in landmark trials. Despite accounting for a smaller proportion of use, patients treated with programmed death-ligand 1 (PD-L1) inhibitors had the highest probability of experiencing a CVD in the FAERS database and the second highest probability in FDA-supporting trials. Given the growing use of immunotherapy in sarcomas, and the lack of general cardiac focused data in this populations, these observations may bear particular relevance [26].

While this investigation corroborates previous findings regarding the cardiotoxicity of cancer therapies, differences in patient populations, treatment modalities, and study designs may account for variations in reported adverse events. Nonetheless, the consistent emergence of cardiovascular adverse events across studies highlights the need for continued research and vigilance in this domain. The clinical relevance of the findings lies in the overall contribution enhancing the understanding of cardiotoxicity in soft tissue sarcoma treatment. The incidence and severity of cardiovascular events associated with novel therapies were quantified and this review provides valuable insights that can inform clinical practice guidelines and patient management strategies. Clinicians must remain vigilant in monitoring patients for cardiovascular symptoms during treatment and consider implementing preemptive cardioprotective measures where appropriate. We suggest monitoring standard electrocardiograms, blood pressure, and cardiac imaging at regular intervals during and after treatment, in line with emerging guidelines [27,28]. For management of hypertension, we suggest treatment in accordance with the current guidelines for the general population [29,30]. For management of arrythmias, we recommend using arrhythmia treatment algorithms similar to the general population with additional considerations for potential drug–drug interactions as detailed in emerging cardio-oncology guidelines [31].

Although the exact reasons for these observations are incompletely understood, several factors may account in part for this. Vascular endothelial growth factor (VEGF) targeted therapies are frequently used in sarcoma [32]. In preclinical and biologic studies, VEGF inhibitors induce substantial cardiac and vascular remodeling, with prevalent hypertension [33,34,35]. Similarly, both VEGF inhibitors and immune checkpoint inhibitors induce arrhythmia and ischemic events in animal and human models [36,37,38,39,40]. With ICI therapies, prior investigations established an increase arrhythmic risk among patients with and without overt myocarditis [38,41,42]. Yet, the exact mechanism(s) for these arrhythmias, particularly in those without myocarditis, require further investigation. Moreover, much of the appreciation of potential cardiotoxicity with these agents was not appreciated until after the onset of several landmark trials. As survival improves, recognition of other unintended or long-term sequelae grows, supporting a higher signal of cardiac risk in the FDA post-marketing dataset, which may not have been appreciated in the cancer efficacy focused trials.

### Limitations

Entries in the FAERS database may be influenced by potential reporting bias due to its voluntary nature. The data from the source neither populate the incidence of adverse events overall nor give a proper indicator for the efficacy of the reported drug. Some of the trials mentioned either have not had their results reported or are still in the active enrollment phase. It is also critical to note that soft tissue sarcoma represents 1% of all cancer diagnoses across the United States [1]. The issues lie within the reported data being either too broad in FAERS or difficult to pinpoint in the publications along with the method of reporting [11]. 

Soft tissue sarcoma also encompasses a variety of sarcoma subtypes with a wide range of underlying molecular mechanisms. The specific targeted therapy or immunotherapy indicated for a particular soft-tissue sarcoma subtype may not be effective in others due to the heterogeneity of their mechanisms. Therefore, the adverse cardiovascular events reported in this study should be viewed within the context of each soft-tissue sarcoma subtype. Furthermore, soft-tissue sarcoma and its subtypes are rare, which is reflected in the small sample sizes of the reviewed studies. The entries from FAERS can be difficult to validate given the limited information that is supplied and may not report every category of cardiovascular-related adverse events.

## 5. Conclusions

The evaluation of cardiotoxic risk associated with novel therapies for soft tissue sarcoma (STS) is imperative due to the increased utilization of these treatments and the increased risk of cardiovascular events. This evaluation highlights the importance of informed decisions in clinical practice through increased knowledge of the cardiovascular risks associated with novel STS treatments.

**Key Points**

- Targeted therapies and immune checkpoint inhibitors for soft tissue sarcoma are associated with adverse cardiovascular events.
- Hypertension and atrial fibrillation were highly reported adverse cardiovascular events in both clinical trials supporting FDA-approval and FAERS.

## Figures and Tables

**Table 1:** Incidence of cardiovascular adverse events for soft-tissue sarcoma FDA-approved targeted and immunotherapies reported in FDA supporting studies.

	Crizotinib **	Imatinib	Pazopanib	Regorafenib †	Ripretinib	Sunitinib ††	Entrectinib	Larotrectinib ‡	Sirolimus	Tazemetostat	Atezolizumab
**Drug Class**											
**Clinical Trial Identifier**	NCT00939770	NCT00085475	NCT00753688	NCT01271712	NCT03353753	NCT00075218	NCT02097810	NCT02122913	NCT02494570	NCT02601950	NCT03141684
							NCT02568267	NCT02637687			
							2012–000148–88	NCT02576431			
**Sample Size**	n = 14	n = 24	n = 240	n = 190	n = 85	n = 483	n = 68	††	n = 34	n = 62	n = 49
Acute coronary syndrome				7				††	1		
Arrhythmia *								††			11
Arrhythmia supraventricular						1		††			
Atrial fibrillation				1		2		††			
Bradycardia	2		5			5		††	1		
Cardiac arrest				1		2		††			
Cardiac failure					1	3	2	††			
Cardiac failure congestive						3		††			
Cardio-respiratory arrest						4		††			
Cardiomegaly						1		††			
Cardiomyopathy						4		††			
Conduction disorder				1				††			
Deep vein thrombosis						13		††			
Ejection fraction decreased						25		††			
Electrocardiogram QT prolonged						1		††			
Electrocardiogram ST segment depression						1		††			
Embolism					1	2		††			
Essential hypertension						1		††			
Haemorrhage						5		††			
Heart failure				1				††			
Hypertension		2	101	125	12	115		††		6	21
Hypertensive crisis						6		††			
Hypotension	4			7		12	15	††	1		
Jugular vein thrombosis						2		††			
Left atrial dilatation						2		††			
Left ventricular dysfunction			19			10		††			
Left ventricular failure						2		††			
Left ventricular hypertrophy						1		††			
Mitral valve imcompetence						2		††			
Myocardial infarction						2		††			
Myocardial ischaemia						1		††			
Orthostatic hypotension						2		††			
Palpitations						4		††			
Pericardial effusion					1	3		††			
Peripheral edema					14	105		††			
Peripheral ischaemia				1		1		††			
Phlebitis						1		††			
Prolonged QT	1							††		1	
Sinus arrhythmia						1		††			
Sinus bradycardia						2		††			
Supraventricular tachycardia						1		††			
Systolic hypertension						1		††			
Tachycardia						7		††	4		
Thromboembolic event				10				††			
Thrombophlebitis						4		††			
Thrombosis						2		††			
Vasculitis						2		††			
Venous thrombosis limb						2		††			
Ventricular dysfunction						1		††			
Ventricular extrasystoles						1		††			
Ventricular hypokinesia						3		††			
Adverse Cardiovascular Events	7	2	125	154	29	371	17	††	7	7	32
**Total Adverse Cardiovascular Events**	**751**										
	**Drug Class Table Legend**										
	Tyrosine kinase inhibitor										
	Tropomyosin receptor kinase inhibitor										
	Mammalian target of rapamycin inhibitor										
	Enhancer of zeste homolog 2 inhibitor										
	Programmed death-ligand 1 inhibitor										

(Arrhythmia *): Includes atrial fibrillation, sinus bradycardia, ventricular tachycardia, and sinus tachycardia. (Crizotinib **): Adverse event results for Crizotinib include data for pediatric patients only. For adult patients, adverse events were reported for all indications combined, so IMT data were not available. (Regorafenib †): Treated with Regorafenib at any time from NCT0127171. (Sunitinib ††): Combined Sunitinib double-blind treatment and Sunitinib open-label treatment from NCT00075218. (Larotrectinib ‡): Adverse event results for Larotrectinib reported a combination of several indications.

**Table 2:** Incidence of cardiovascular adverse events for soft-tissue sarcoma FDA-approved targeted and immunotherapies from FDA adverse events reporting system (FAERS). Abbreviations: FAERS, FDA adverse events reporting system.

	Crizotinib *	Imatinib **	Pazopanib	Regorafenib	Ripretinib	Sunitinib	Entrectinib	Larotrectinib	Sirolimus	Tazemetostat	Atezolizumab
**Drug Class**											
**Sample Size**	-	-	n = 951	n = 425	n = 3132	n = 60	n = 1	n = 7	n = 11	n = 17	n = 6
Arrhythmia	-	-	2	0	0	0	0	0	0	0	0
Atrial fibrillation	-	-	2	9	17	2	0	0	0	0	1
Cardiac arrest	-	-	3	0	3	0	0	0	0	0	0
Cardiac failure	-	-	8	6	5	3	0	0	0	0	1
Cardiac flutter	-	-	0	0	1	0	0	0	0	0	0
Cardiac tamponade	-	-	1	0	0	0	0	0	0	0	0
Cardio-respiratory arrest	-	-	0	0	1	1	0	0	0	0	0
Cardiogenic shock	-	-	1	0	0	2	0	0	0	0	0
Cardiomegaly	-	-	0	1	1	0	0	0	0	0	0
Cardiomyopathy	-	-	1	4	3	0	0	0	0	0	0
Congestive cardiac failure	-	-	1	1	4	1	0	0	0	0	0
Coronary artery disease	-	-	0	1	4	0	0	0	0	0	0
Coronary artery occlusion	-	-	0	0	1	0	0	0	0	0	0
Coronary artery stensosis	-	-	0	1	1	0	0	0	0	0	0
Decreased blood pressure	-	-	3	0	0	0	0	0	0	0	0
Edema	-	-	1	1	6	0	0	0	0	0	0
Ejection fraction decreased	-	-	2	0	1	1	0	0	0	0	0
EKG abnormality	-	-	3	0	0	0	0	0	0	0	0
Hypertension	-	-	36	53	175	8	0	1	0	0	2
Hypotension	-	-	2	3	15	1	0	0	0	0	0
Left ventricular dysnfunction	-	-	0	1	2	0	0	0	0	0	0
Left ventricular hypertrophy	-	-	0	1	0	0	0	0	0	0	0
Myocardial infarction	-	-	3	3	9	0	0	0	0	0	0
Myocardial iscaemia	-	-	0	2	0	0	0	0	0	0	0
Orbital edema	-	-	0	0	1	0	0	0	0	0	0
Periorbital edema	-	-	0	0	0	0	0	0	0	0	0
Peripheral edema	-	-	6	3	12	1	0	0	1	1	0
Pulmonary edema	-	-	4	0	4	0	0	0	0	0	0
Pulmonary embolism	-	-	5	3	5	0	0	0	0	0	1
Tachycardia	-	-	1	2	4	0	0	0	0	0	1 †
Transient ischaemic attack	-	-	0	1	3	0	0	0	0	0	0
Ventricular tachycardia	-	-	0	0	0	0	0	0	0	0	1
Adverse Events (Any)	-	-	2159	2161	7729	151	1	21	29	53	33
Adverse Cardiovascular Events	-	-	85	96	278	20	0	1	1	1	7
Total Adverse Events (Any)	12,337										
**Total Adverse Cardiovascular Events**	**489**										
	**Drug Class Table Legend**										
	Tyrosine kinase inhibitor										
	Tropomyosin receptor kinase inhibitor										
	Mammalian target of rapamycin inhibitor										
	Enhancer of zeste homolog 2 inhibitor										
	Programmed death-ligand 1 inhibitor										

(Crizotinib *): No reason for use that includes ALK positive. (Imatinib **): No reason for use that includes dermatofibrosarcoma protuberans. †: Sinus tachycardia.

## Data Availability

The data that support the findings of this study are not openly available due to reasons of sensitivity and are available from the corresponding author upon reasonable request.

## Supplementary Materials

The following supporting information can be downloaded at: https://www.mdpi.com/article/10.3390/cancers17050827/s1, Table S1: FDA-approved targeted therapies and immunotherapies for soft-tissue sarcoma; Table S2: Comparative incidence of cardiovascular adverse events between treatment and placebo arms of FDA supporting studies for soft-tissue sarcoma FDA-approved targeted and immunotherapies; Table S3: (A) Clinical trial designs; (B) Landmark sarcoma trials, supporting food and drug administration (FDA) approved therapies.
