# Supplementary material for: Characterizing Cardiotoxicity of FDA-Approved Soft Tissue Sarcoma Targeted Therapies and Immune Checkpoint Inhibitors: A Systematic Review
Source: Cancers (Basel). 2025 Feb 27;17(5):827. doi: 10.3390/cancers17050827 (PMC11899116; doi:10.3390/cancers17050827)
Supplement: Supplementary file 1 [file cancers-17-00827-s001.zip › cancers-3439781-supplementary.pdf]

**Supplemental Table S1.** FDA-approved targeted therapies and immunotherapies for soft-tissue sarcoma.

| | Therapy Name | Indication | Target |
|---|---|---|---|
| **FDA Approved Targeted Therapies** | Tazemetostat | Epithelioid Sarcoma | EZH2 Inhibitor |
| | nab-Sirolimus | Perivascular Epithelioid Cell Tumor (PEComa) | mTOR inhibitor |
| | Entrectinib | NTRK Gene Fusion-Positive Solid Tumors | TRK inhibitor |
| | Larotrectinib | NTRK Gene Fusion-Positive Solid Tumors | TRK inhibitor |
| | Crizotinib | ALK-Positive Inflammatory Myofibroblastic Tumor | Tyrosine Kinase Inhibitor |
| | Imatinib | Dermatofibrosarcoma Protuberans | Tyrosine Kinase Inhibitor |
| | Pazopanib | Soft Tissue Sarcoma | Tyrosine Kinase Inhibitor |
| | Regorafenib | Gastrointestinal Stromal Tumor (GIST) | Tyrosine Kinase Inhibitor |
| | Ripretinib | Gastrointestinal Stromal Tumor (GIST) | Tyrosine Kinase Inhibitor |
| | Sunitinib | Gastrointestinal Stromal Tumor (GIST) | Tyrosine Kinase Inhibitor |
| **FDA Approved Immunotherapies** | Atezolizumab | Alveolar Soft Part Sarcoma | PD-L1 Inhibitor |

**Abbreviations:** ALK: anaplastic lymphoma kinase; EZH2: enhancer of zeste homolog 2; GIST: gastrointestinal stromal tumor; mTOR: mammalian target of rapamycin; NTRK: neurotrophic tyrosine receptor kinase; PD-L1: programmed death-ligand 1; PEComa: perivascular epithelioid cell tumor; TRK: tropomyosin receptor kinase.

**Supplemental Table S2.** Comparative incidence of cardiovascular adverse events between treatment and placebo arms of FDA supporting studies for soft-tissue sarcoma FDA-approved targeted and immunotherapies.

| | **Pazopanib** | | **Regorafenib †** | | **Ripretinib** | | **Sunitinib ††** | |
|---|---|---|---|---|---|---|---|---|
| **Drug Class** | Tyrosine Kinase Inhibitor | | Tyrosine Kinase Inhibitor | | Tyrosine Kinase Inhibitor | | Tyrosine Kinase Inhibitor | |
| **Clinical Trial Identifier** | NCT00753688 | | NCT01271712 | | NCT03353753 | | NCT00075218 | |
| **Treatment Group** | Placebo | Pazopanib | Placebo | Regorafenib | Placebo | Ripretinib | Placebo | Sunitinib |
| **Sample Size** | n = 123 | n = 240 | n = 8 | n = 190 | n = 43 | n = 85 | n = 114 | n = 483 |
| Acute coronary syndrome | | | 0 | 7 | | | | |
| Arrhythmia * | | | | | | | | |
| Arrhythmia supraventricular | | | | | | | 0 | 1 |
| Atrial fibrillation | | | 0 | 1 | | | 1 | 2 |
| Bradycardia | ‡ | 5 | | | | | 0 | 5 |
| Cardiac arrest | | | 0 | 1 | | | 2 | 2 |
| Cardiac failure | | | | | 0 | 1 | 2 | 3 |
| Cardiac failure congestive | | | | | | | 0 | 3 |
| Cardio-respiratory arrest | | | | | | | 0 | 4 |
| Cardiomegaly | | | | | | | 0 | 1 |
| Cardiomyopathy | | | | | | | 0 | 4 |
| Conduction disorder | | | 0 | 1 | | | | |
| Deep vein thrombosis | | | | | | | 0 | 13 |
| Ejection fraction decreased | | | | | | | 3 | 25 |
| Electrocardiogram QT prolonged | | | | | | | 0 | 1 |
| Electrocardiogram ST segment depression | | | | | | | 0 | 1 |
| Embolism | | | | | 0 | 1 | 0 | 2 |
| Essential hypertension | | | | | | | 0 | 1 |
| Haemorrhage | | | | | | | 1 | 5 |
| Heart failure | | | 0 | 1 | | | | |
| Hypertension | 7 | 101 | 3 | 125 | 2 | 12 | 9 | 115 |
| Hypertensive crisis | | | | | | | 0 | 6 |
| Hypotension | | | 0 | 7 | | | 1 | 12 |
| Jugular vein thrombosis | | | | | | | 0 | 2 |
| Left atrial dilatation | | | | | | | 1 | 2 |
| Left ventricular dysfunction | 5 | 19 | | | | | 1 | 10 |
| Left ventricular failure | | | | | | | 0 | 2 |
| Left ventricular hypertrophy | | | | | | | 0 | 1 |
| Mitral valve imcompetence | | | | | | | 0 | 2 |
| Myocardial infarction | | | | | | | 0 | 2 |
| Myocardial ischaemia | | | | | | | 0 | 1 |
| Orthostatic hypotension | | | | | | | 0 | 2 |
| Palpitations | | | | | | | 2 | 4 |
| Pericardial effusion | | | | | 0 | 1 | 0 | 3 |
| Peripheral edema | | | | | 3 | 14 | 15 | 105 |
| Peripheral ischaemia | | | 0 | 1 | | | 0 | 1 |
| Phlebitis | | | | | | | 0 | 1 |
| Prolonged QT | | | | | | | | |
| Sinus arrhythmia | | | | | | | 0 | 1 |
| Sinus bradycardia | | | | | | | 0 | 2 |
| Supraventricular tachycardia | | | | | | | 0 | 1 |
| Systolic hypertension | | | | | | | 0 | 1 |
| Tachycardia | | | | | | | 0 | 7 |
| Thromboembolic event | | | 2 | 10 | | | | |
| Thrombophlebitis | | | | | | | 0 | 4 |
| Thrombosis | | | | | | | 0 | 2 |
| Vasculitis | | | | | | | 0 | 2 |
| Venous thrombosis limb | | | | | | | 0 | 2 |
| Ventricular dysfunction | | | | | | | 0 | 1 |
| Ventricular extrasystoles | | | | | | | 0 | 1 |
| Ventricular hypokinesia | | | | | | | 0 | 3 |
| Adverse Cardiovascular Events (ACE) | 12 | 125 | 5 | 154 | 5 | 29 | 38 | 371 |
| Probability to Experience ACE | 0.10 | 0.52 | 0.63 | 0.81 | 0.12 | 0.34 | 0.33 | 0.77 |
| Probability to Experience ACE Ratio (Treatment/Control) | 5.34 | | 1.30 | | 2.93 | | 2.30 | |
| Total Probability to Experience ACE in Placebo | 0.21 | | | | | | | |
| Total Probability to Experience ACE in Treatment | 0.68 | | | | | | | |
| **Total Probability to Experience ACE Ratio (Treatment/Control)** | **3.27** | | | | | | | |

‡: No data reported for bradycardia in Pazopanib placebo arm.

(Regorafenib †): Treated with Regorafenib at any time from NCT0127171.

(Sunitinib ††): Combined Sunitinib double-blind treatment and Sunitinib open-label treatment from NCT00075218.

**Supplemental Table S3A.** Clinical trial designs.

| Drug | Clinical Trial Design |
|---|---|
| Crizotinib | NCT00939770 was a multicenter, single-arm, open-label study. |
| Imatinib | NCT00084630 and NCT00085475 were single-agent, single-arm, open-label, multicenter phase II clinical trials. |
| Pazopanib | NCT00753688 was a randomized, double-blind, placebo-controlled, multicenter trial. |
| Regorafenib | NCT01271712 was a multicenter, randomized (2:1), double-blind, placebo-controlled trial. |
| Ripretinib | NCT03353753 was a multi-center, randomized (2:1), double-blind, placebo-controlled trial. |
| Sunitinib | NCT00075218 was a 2-arm, international, randomized, double-blind, placebo-controlled trial. |
| Entrectinib | NCT02097810, NCT02568267, and 2012–000148–88 were multicenter, single-arm, open-label clinical trials. |
| Larotrectinib | NCT02122913, NCT02637687, and NCT02576431 were multicenter, open-label, single-arm clinical trials. |
| Sirolimus | NCT02494570 was a multi-center, single-arm clinical trial. |
| Tazemetostat | NCT02601950 was an open-label, single-arm cohort of a multi-center study. |
| Atezolizumab | NCT03141684 was an open-label, single-arm study. |

**Supplemental Table S3B**. Landmark sarcoma trials, supporting food and drug administration (FDA) approved therapies.

| Drug | NCT # | Landmark trial Citation |
|---|---|---|
| Crizotinib | NCT00939770 | Mossé YP, Voss SD, Lim MS, Rolland D, Minard CG, Fox E, Adamson P, Wilner K, Blaney SM, Weigel BJ. Targeting ALK With Crizotinib in Pediatric Anaplastic Large Cell Lymphoma and Inflammatory Myofibroblastic Tumor: A Children's Oncology Group Study. *J Clin Oncol*. 2017 Oct 1;35(28):3215–3221. doi: 10.1200/JCO.2017.73.4830. Epub 2017 Aug 8. PMID: 28787259; PMCID: PMC5617123. |
| Imatinib | NCT00084630 and NCT00085475 | Rutkowski P, Van Glabbeke M, Rankin CJ, Ruka W, Rubin BP, Debiec-Rychter M, Lazar A, Gelderblom H, Sciot R, Lopez-Terrada D, Hohenberger P, van Oosterom AT, Schuetze SM; European Organisation for Research and Treatment of Cancer Soft Tissue/Bone Sarcoma Group; Southwest Oncology Group. Imatinib mesylate in advanced dermatofibrosarcoma protuberans: pooled analysis of two phase II clinical trials. *J Clin* |

| | | |
|---|---|---|
| | | *Oncol*. 2010 Apr 1;28(10):1772–9. doi: 10.1200/JCO.2009.25.7899. Epub 2010 Mar 1. PMID: 20194851; PMCID: PMC3040044. |
| Pazopanib | NCT00753688 | van der Graaf WT, Blay JY, Chawla SP, Kim DW, Bui-Nguyen B, Casali PG, Schöffski P, Aglietta M, Staddon AP, Beppu Y, Le Cesne A, Gelderblom H, Judson IR, Araki N, Ouali M, Marreaud S, Hodge R, Dewji MR, Coens C, Demetri GD, Fletcher CD, Dei Tos AP, Hohenberger P; EORTC Soft Tissue and Bone Sarcoma Group; PALETTE study group. Pazopanib for metastatic soft-tissue sarcoma (PALETTE): a randomised, double-blind, placebo-controlled phase 3 trial. *Lancet*. 2012 May 19;379(9829):1879–86. doi: 10.1016/S0140-6736(12)60651-5. Epub 2012 May 16. PMID: 22595799. |
| Regorafenib | NCT01271712 | Demetri GD, Reichardt P, Kang YK, Blay JY, Rutkowski P, Gelderblom H, Hohenberger P, Leahy M, von Mehren M, Joensuu H, Badalamenti G, Blackstein M, Le Cesne A, Schöffski P, Maki RG, Bauer S, Nguyen BB, Xu J, Nishida T, Chung J, Kappeler C, Kuss I, Laurent D, Casali PG; GRID study investigators. Efficacy and safety of regorafenib for advanced gastrointestinal stromal tumours after failure of imatinib and sunitinib (GRID): an international, multicentre, randomised, placebo-controlled, phase 3 trial. *Lancet*. 2013 Jan 26;381(9863):295–302. doi: 10.1016/S0140-6736(12)61857-1. Epub 2012 Nov 22. PMID: 23177515; PMCID: PMC3819942. |
| Ripretinib | NCT03353753 | Blay JY, Serrano C, Heinrich MC, Zalcberg J, Bauer S, Gelderblom H, Schöffski P, Jones RL, Attia S, D'Amato G, Chi P, Reichardt P, Meade J, Shi K, Ruiz-Soto R, George S, von Mehren M. Ripretinib in patients with advanced gastrointestinal stromal tumours (INVICTUS): a double-blind, randomised, placebo-controlled, phase 3 trial. *Lancet Oncol*. 2020 Jul;21(7):923–934. doi: 10.1016/S1470-2045(20)30168-6. Epub 2020 Jun 5. Erratum in: Lancet Oncol. 2020 Jul;21(7):e341. doi: 10.1016/S1470-2045(20)30353-3. PMID: 32511981; PMCID: PMC8383051. |
| Sunitinib | NCT00075218 | Demetri GD, van Oosterom AT, Garrett CR, Blackstein ME, Shah MH, Verweij J, McArthur G, Judson IR, Heinrich MC, Morgan JA, Desai J, Fletcher CD, George S, Bello CL, Huang X, Baum CM, Casali PG. Efficacy and safety of sunitinib in patients with advanced gastrointestinal stromal tumour after failure of imatinib: a randomised controlled trial. *Lancet*. 2006 Oct 14;368(9544):1329–38. doi: 10.1016/S0140-6736(06)69446-4. PMID: 17046465. |
| Entrectinib | NCT02097810, NCT02568267, and 2012–000148–88 | Doebele RC, Drilon A, Paz-Ares L, Siena S, Shaw AT, Farago AF, Blakely CM, Seto T, Cho BC, Tosi D, Besse B, Chawla SP, Bazhenova L, Krauss JC, Chae YK, Barve M, Garrido-Laguna I, Liu SV, Conkling P, John T, Fakih M, Sigal D, Loong HH, Buchschacher GL Jr, Garrido P, Nieva J, Steuer C, Overbeck TR, Bowles DW, Fox E, Riehl T, Chow-Maneval E, Simmons B, Cui N, Johnson A, Eng S, Wilson TR, Demetri GD; trial investigators. |

| | | |
|---|---|---|
| | | Entrectinib in patients with advanced or metastatic NTRK fusion-positive solid tumours: integrated analysis of three phase 1–2 trials. *Lancet Oncol*. 2020 Feb;21(2):271–282. doi: 10.1016/S1470-2045(19)30691-6. Epub 2019 Dec 11. Erratum in: Lancet Oncol. 2020 Feb;21(2):e70. doi: 10.1016/S1470-2045(20)30029-2. Erratum in: Lancet Oncol. 2020 Jul;21(7):e341. doi: 10.1016/S1470-2045(20)30345-4. Erratum in: Lancet Oncol. 2020 Aug;21(8):e372. doi: 10.1016/S1470-2045(20)30382-X. Erratum in: Lancet Oncol. 2021 Oct;22(10):e428. doi: 10.1016/S1470-2045(21)00538-6. PMID: 31838007; PMCID: PMC7461630. |
| Larotrectinib | NCT02122913, NCT02637687, and NCT02576431 | Hong DS, DuBois SG, Kummar S, Farago AF, Albert CM, Rohrberg KS, van Tilburg CM, Nagasubramanian R, Berlin JD, Federman N, Mascarenhas L, Geoerger B, Dowlati A, Pappo AS, Bielack S, Doz F, McDermott R, Patel JD, Schilder RJ, Tahara M, Pfister SM, Witt O, Ladanyi M, Rudzinski ER, Nanda S, Childs BH, Laetsch TW, Hyman DM, Drilon A. Larotrectinib in patients with TRK fusion-positive solid tumours: a pooled analysis of three phase 1/2 clinical trials. *Lancet Oncol*. 2020 Apr;21(4):531–540. doi: 10.1016/S1470-2045(19)30856-3. Epub 2020 Feb 24. PMID: 32105622; PMCID: PMC7497841. |
| Sirolimus | NCT02494570 | Wagner AJ, Ravi V, Riedel RF, Ganjoo K, Van Tine BA, Chugh R, Cranmer L, Gordon EM, Hornick JL, Du H, Grigorian B, Schmid AN, Hou S, Harris K, Kwiatkowski DJ, Desai NP, Dickson MA. *nab*-Sirolimus for Patients With Malignant Perivascular Epithelioid Cell Tumors. *J Clin Oncol*. 2021 Nov 20;39(33):3660–3670. doi: 10.1200/JCO.21.01728. Epub 2021 Oct 12. Erratum in: J Clin Oncol. 2023 Dec 10;41(35):5477. doi: 10.1200/JCO.23.02173. PMID: 34637337; PMCID: PMC8601264. |
| Tazemetostat | NCT02601950 | Gounder M, Schöffski P, Jones RL, Agulnik M, Cote GM, Villalobos VM, Attia S, Chugh R, Chen TW, Jahan T, Loggers ET, Gupta A, Italiano A, Demetri GD, Ratan R, Davis LE, Mir O, Dileo P, Van Tine BA, Pressey JG, Lingaraj T, Rajarethinam A, Sierra L, Agarwal S, Stacchiotti S. Tazemetostat in advanced epithelioid sarcoma with loss of INI1/SMARCB1: an international, open-label, phase 2 basket study. *Lancet Oncol*. 2020 Nov;21(11):1423–1432. doi: 10.1016/S1470-2045(20)30451-4. Epub 2020 Oct 6. PMID: 33035459. |
| Atezolizumab | NCT03141684 | Chen AP, Sharon E, O'Sullivan-Coyne G, Moore N, Foster JC, Hu JS, Van Tine BA, Conley AP, Read WL, Riedel RF, Burgess MA, Glod J, Davis EJ, Merriam P, Naqash AR, Fino KK, Miller BL, Wilsker DF, Begum A, Ferry-Galow KV, Deshpande HA, Schwartz GK, Ladle BH, Okuno SH, Beck JC, Chen JL, Takebe N, Fogli LK, Rosenberger CL, Parchment RE, Doroshow JH. Atezolizumab for Advanced Alveolar Soft Part Sarcoma. *N Engl J Med*. 2023 Sep 7;389(10):911–921. doi: 10.1056/NEJMoa2303383. PMID: 37672694; PMCID: PMC10729808. |
